# Travelling for Health

**Published:** 1869-11

**Authors:** 


### TRAVELLING FOR HEALTH.

My Dear Doctor:

If you and your readers will indulge me once more, I will ransack my budget of invalid memories, for a brief experimental chapter on Liver Complaint, hoping that some poor luckless wight, who thinks he knows more than a hundred doctors, may suspect his intrinsic stupidity in time to cheat the undertaker out of a premature fee. So much for the exordium, now for the sermon.

About the first of February, 1857, while spending the winter in Florida, I was suddenly attacked with a most unaccountable expectoration of mucus. I was continually hawking it up from morning till night; and the result was, that in less than a fortnight, my physical vigor had almost left me, and I appeared to myself a fair candidate for an early death. I went to a doctor, with a pain in my right lung, and he prescribed cupping, and was proceeding to execute, when his patient went off in a fainting fit, and he desisted.

For the space of about six months, this torrent of expectoration continued, with the most obstinate constipation, and an utter prostration of spirits, till, on my return to the North, my case seemed about beyond the reach of remedy. It is unnecessary to enter into detail. Suffice it to say, that mine was one of the worst cases of Liver Complaint that ever I heard of.

Now for the philosophy. I went to Florida for the cure of a cough, being, as I supposed, in a "decline." My family physician said: "Take cod liver oil;" and I took enough to carbonize a whale. Everybody at the South said: "Eat sugar cane;" "Eat fat meat;" and didn't I obey "everybody?" I bought me about half a cord of sugar canes from a neighboring plantation, and chewed at the rate of fifteen feet a day, swallowing the juice. "Everybody" said: "People in sugar houses never had coughs," and so I went to sugar mills, and breathed in the hot steam from sugar boilers by the hour. "Everybody" never told me where all the oxygen was coming from, in a Southern air, to burn up all this immense quantity of carbon. But, no matter for that. As for fat meat, I ate spoiled bacon enough to make an Esquimaux dream of scrofula.

"Everybody" said exercise was a good thing, and you would think I believed it, to have seen a minister having bid farewell to sermons and people, with his shirt sleeves rolled up, swinging an axe upon Florida pines, six or eight hours a day, with the thermometer at 85° to 90° Fahrenheit. I kept myself in a *continuous sweat* for about three months; and, about the first of February, as aforesaid, desisted from active exercise, intending to complete a cure by a thousand miles travel on horseback. As I look back upon it now, I do not doubt that my disease was caused, in the main, by that excessive perspiration, followed by a sudden checking of it, in a suspension of violent physical exercise. Put this, and cod liver oil, and sugar drippings, and ham-rinds all together, and if Goliath of Gath wouldn't have Liver Complaint, then the laws of nature are out of course.

How many another dark chapter, in the confessions of an invalid, could I give; but I spare your readers. Will you admit the truth of three sequences from this rambling sermon?

1. Never take more carbon into the system than you have oxygen to burn.

2. Never suddenly suspend the action of any of the depurating functions of the body, the bowels and skin especially.

3. When you are sick, don't be so stupid as to think that you know more than a college of educated physicians, and all the universe besides. As ever, yours,

LEONARD.

